# Supplementary material for: Integrating Aggressive-Variant Prostate Cancer-Associated Tumor Suppressor Gene Status with Clinical Variables to Refine Prognosis and Predict Androgen Receptor Pathway Inhibitor Response in Metastatic Hormone-Sensitive Setting
Source: Int J Mol Sci. 2025 May 31;26(11):5309. doi: 10.3390/ijms26115309 (PMC12154572; doi:10.3390/ijms26115309)
Supplement: Supplementary file 1 [file ijms-26-05309-s001.zip › ijms-3631138-supplementary.pdf]

International Journal of *Molecular Sciences*

MDPI

Supplementary Materials

Supplementary Materials

Method S1 - Inclusion Criteria

Method S2 – Exclusion Criteria

Method S3 – Tumor specimens and targeted sequencing

Method S4 – Collected parameters and informed consent

Method S5 – Data Extraction Process

Method S6 – Secondary Objectives and exploratory analysis

Method S7 – Integration of AVPC-TSG status within clinical variables

Method S8 - Statistical analyses

Method S9 - Exploratory analysis and sensitivity analysis

Results S1 - Modified ESMO-GROW flowchart for real-world evidence studies in oncology

Table S1 - Site of Analysed Tissue Samples

Table S2 - Type and Frequency of detected gene alterations in the overall population

Table S3 –Univariate and multivariate Cox proportional hazard models for PFS including site of metastasis and oligometastatic disease status

Table S4 - Univariate and multivariate Cox proportional hazard models for OS including site of metastasis and oligometastatic disease status

Table S5 –Sensitivity Analysis for PFS: Univariate and multivariate Cox proportional hazard models for PFS

Table S6 - Sensitivity Analysis for OS: Univariate and multivariate Cox proportional hazard models for OS

Table S7 – Exploratory Analysis: Univariate and multivariate Cox proportional hazard models for PFS and OS based on Disease Presentation

Table S8 Exploratory Analysis: Univariate Cox proportional hazard models for PFS and OS based on AVPC-TSGalt number

Table S9 – Integrating TSG Status with Chaarted volume criteria: Univariate Cox Analysis for PFS and OS and Pairwise Comparison

Table S10 - Type of I Line Treatment Based on AVPC-TSG Status and Chaarted Disease Volume Status

Table S11. Univariate Cox proportional hazard models for PFS and OS comparing "Intermediate Risk Subgroup" components (AVPC-TSGalt/LV VS AVPC-TSGwt/HV)

Table S12 – Integrating TSG Status with ISUP Grade: Univariate Cox Analysis for PFS and OS and Pairwise Comparison

Table S13. Integrating TSG Status with Disease Presentation: Univariate Cox Analysis for PFS and OS and Pairwise Comparison

Table S14 – Integrating disease presentation and ISUP Grade Group with Chaarted volume criteria: Univariate Cox Analysis for PFS and Pairwise Comparison

Table S15. Univariate Cox proportional hazard models for PFS based on first line treatment in the overall population and in AVPC-TSGalt and AVPC-TSGwt patients

Table S16. Exploratory Analysis: Univariate Cox proportional hazard models for PFS based on first line treatment in subgroups defined by AVPC-TSG status and CHAARTED volume criteria

Table S17. Exploratory Analysis: Univariate Cox proportional hazard models for PFS based on first line treatment in the "intermediate risk subgroup" components

Table S18. Self-reported ESMO-GROW informative Score
Figure S1. Forest Plot of MV Cox Analysis for PFS and OS with relevant clinical variables
Figure S2. Integrating AVPC-TSG Status with Disease Volume and ISUP Grade, Kaplan-Meier Curves for PFS and OS

Method S1 - Inclusion Criteria

Inclusion criteria:

To be included in the study, patients had to present the following:

- Males of 21 years of age and above
- Histological or cytological confirmed diagnosis of PC from prostate biopsy, radical prostatectomy or transurethral resection of the prostate (TURP) or biopsy from a metastatic site
- Metastatic disease (M1a, b or c stage as defined by the American Joint Committee on Cancer)
- Radiologic evidence of metastases at conventional imaging (de novo or metachronous mHSPC)
- Included patients presented with an Eastern Cooperative Oncology Group (ECOG) performance status score of 0,1 or 2.
- All men received pharmacological androgen deprivation therapy, with a serum testosterone level of 50 ng/dl or less (≤1.7 nmol/L)
- First line treatment with either ADT monotherapy or in association with docetaxel and/or ARPI.
- Tissue NGS analysis already performed OR availability of archival formalin-fixed, paraffin-embedded tumor tissue from a prostate biopsy and/or prostatectomy or other diagnostic biopsies obtained before the initiation of systemic treatment for metastatic prostate cancer.

Method S2 – Exclusion Criteria

Exclusion criteria:

- Primary pure small cell carcinoma of the prostate
- Other malignancy that required cytotoxic therapy simultaneously
- Tumor tissue specimen unavailable or patient deceased before tissue NGS analysis
- Missing data to an extent that none of the research objectives could be addressed

Method S3 – Tumor specimens and targeted sequencing

All patients had archival formalin-fixed, paraffin-embedded (FFPE) tumor tissue from a prostate biopsy and/or prostatectomy or other diagnostic biopsies obtained before the initiation of treatment (**sTable1**). Clinical-grade next generation sequencing (NGS) of FFPE tissue from biopsies of patients with metastatic disease was performed by Ion Torrent (Thermo Fisher Scientific, Waltham, MA) using Oncomine Comprehensive Assay v3 (Thermo Fisher Scientific, Waltham, MA). The Oncomine Comprehensive Assay v3 is a multi-biomarker NGS assay that covers 161 cancer-related genes, including TP53, PTEN, AKT, PIK3C/R and RB1, of interest in this study. The test allows the identification of point mutations, insertions and deletions of short nucleotide regions (indels), and variations in gene copy number (CNV) but not other alterations such as, for example, translocations and LOH. The presence of any significant alteration in TP53 and/or PTEN and/or AKT and/or PIK3 and/or RB1 was classified as "AVPC related genes alteration" (AVPC-RGalt). Variants of unknown significance were not included in this analysis.

Method S4 – Collected parameters and informed consent

Progression free survival (PFS) was defined as the time from the start of therapy for mHSPC to identification of radiographic progression, symptoms, initiation of a new treatment, or death, whichever occurred first.

Overall survival (OS) was defined as the time from start of therapy until death of any cause.

Several demographic and clinical parameters were collected to account for potential factors influencing PFS and OS in the multivariate Cox analysis (Supplementary materials):

- Gleason score, ISUP grade.
- Age
- Baseline PSA
- Type of mHSPC treatment
- Type of first line ARPI.
- Volume of the disease (as per CHAARTED criteria, defined on conventional imaging): low or high.
- Type of disease presentation: metachronous or de novo
- Radiotherapy to the prostate bed in the metastatic setting
- Presence of bone metastasis
- Presence of liver metastasis
- Presence of lung metastasis
- Oligometastatic disease (defined as the presence of less than 4 metastatic lesions, excluding limphonodes and visceral metastasis)
- PTEN/PI3K/AKT pathway alteration on next generation sequencing analysis
- RB1 alteration on next generation sequencing analysis
- TP53 alteration on next generation sequencing analysis
- AVPC-TSG alt: defined as the presence of at least one alteration in PTEN/PI3K/AKT pathway, RB1 or TP53 genes on next generation sequencing analysis.
- AVPC-TSG wt: defined as the absence of any alteration in PTEN/PI3K/AKT pathway, RB1 or TP53 genes on next generation sequencing analysis

Informed consent:

Every patient either signed an informed consent for data collection or was deceased at the time of data analysis. The study was in the scope of a retrospective data collection protocol approved by the local ethical committee and was in accordance with the 1964 Helsinki Declaration and its later amendments or comparable ethical standards.

Method S5 – Data Extraction Process

The selection of patients and collection of clinical variables were meticulously carried out through manual processes of two co-authors, who reviewed patient records and systematically extracted relevant clinical, demographical and genomic data. The data extraction was conducted with approval from the ethics committee.

Method S6 – Secondary Objectives and exploratory analysis
Our primary objectives were:

- To investigate the impact of AVPC-TSG alteration status on PFS, in conjunction with established demographical and clinical prognostic variables (CHAARTED volume criteria, type of metastatic disease presentation, ISUP grade) in the overall population.
- To identify patients at very high and very low risk of progression and death by integrating TSG alteration status with clinical variables.
- To test the potential of AVPC-alt status in predicting PFS benefit from ARPIs

Our secondary objectives were:

- To investigate the prognostic impact of AVPC-TSG alteration status in terms of OS together with established clinical prognostic variables (CHAARTED Volume criteria, type of metastatic presentation, ISUP Grade) in the overall population.
- To investigate the prognostic impact of AVPC-TSG alteration status, CHAARTED volume criteria, type of disease presentation and ISUP Grade in terms of PFS and OS together with site of metastasis (liver, lung) and oligometastatic presentation in the overall population.

We conducted exploratory analyses to examine:

- the influence of AVPC-TSG status on PFS and OS in the de novo and relapsed patients populations, separately.
- the impact of first line ARPI across the patients subgroup defined based on the integrations of AVPC-TSG status with clinical variables (CHAARTED volume criteria, type of disease presentation, ISUP Grade).

Method S7 – Integration of AVPC-TSG status within clinical variables

- To provide a more comprehensive analysis and to identify patients at very high risk and very low risk of progression and death, we studied the differential impact of TSG alteration status by incorporating it into clinical variables. Specifically, we created the following subgroup variables:
- AVPC-TSGalt + CHAARTED High Volume (TSGalt/HV) versus AVPC-TSGwt + CHAARTED Low Volume (AVPC-TSGwt/LV) versus only one high-risk feature (AVPC-TSGalt/LV or AVPC-TSGwt/HV)
- AVPC-TSGalt + De Novo (AVPC-TSGalt/DN) presentation versus AVPC-TSGwt + Relapsed (AVPC-TSGwt/R) presentation versus only one high-risk feature (AVPC-TSGalt/R or AVPC-TSGwt/DN)
- AVPC-TSGalt + ISUP Grade 5 (AVPC-TSGalt/IG5) versus AVPC-TSGwt + ISUP Grade <5 (AVPC-TSGwt/IG<5) versus only one high-risk feature (AVPC-TSGalt/IG<5 or AVPC-TSGalt/IG5)

Method S8 - Statistical analyses

Pearson's chi-square test was utilized to examine the association between categorical variables and the Kruskal–Wallis rank sum test for continuous measures, specifically comparing patients whose tumors harbored AVPC-TSG alteration to those without.

The association between AVPC-TSG status and PFS or OS was depicted using Kaplan–Meier survival curves, and group comparisons were made using the log-rank test. Patients without a documented event were censored at their last follow-up. All statistical comparisons were made with two-tailed tests (10).

Univariate analyses were performed employing Cox proportional hazards models, incorporating AVPC-TSGalt status and baseline clinical characteristics as independent variables. Thereafter, variables that demonstrated statistical significance ($P < 0.10$) in the univariate analyses were integrated as covariates in the multivariate Cox model.

Multivariable Cox analysis was performed to assess AVPC-TSG status alongside established prognostic demographic and clinical variables (CHAARTED volume criteria, de novo/relapsed presentation, ISUP grade, age). Interaction tests were conducted to determine whether AVPC-TSG status was independently correlated with PFS and OS.

To provide a more comprehensive analysis and to identify patients at very high risk and very low risk of progression and death, we studied the differential impact of TSG alteration status by incorporating it into clinical variables. Specifically, we created the following subgroup variables:

- AVPC-TSGalt + CHAARTED High Volume (TSGalt/HV) versus AVPC-TSGwt + CHAARTED Low Volume (AVPC-TSGwt/LV) versus only one high-risk feature (AVPC-TSGalt/LV or AVPC-TSGwt/HV)
- AVPC-TSGalt + De Novo (AVPC-TSGalt/DN) presentation versus AVPC-TSGwt + Relapsed (AVPC-TSGwt/R) presentation versus only one high-risk feature (AVPC-TSGalt/R or AVPC-TSGwt/DN)
- AVPC-TSGalt + ISUP Grade 5 (AVPC-TSGalt/IG5) versus AVPC-TSGwt + ISUP Grade <5 (AVPC-TSGwt/IG<5) versus only one high-risk feature (AVPC-TSGalt/IG<5 or AVPC-TSGalt/IG5)

To compare these subgroups, we employed the Holm correction method, a statistical technique used to control the family-wise error rate when performing multiple comparisons. This method sequentially adjusts the p-values to ensure that the overall type I error rate is maintained at a desired level, thereby providing a more stringent criterion for statistical significance (11).

Univariate cox analysis for PFS were performed in the overall population and in AVPC-TSGalt and AVPC-TSGwt populations separately to study de impact of AVPC-TSGalt status on first line treatment with ARPI combinations. The

compared groups were “ARPI combinations” (including patients treated with ADT+ARPI or ADT+ARPI+Docetaxel) versus “ADT or ADT+Docetaxel” (including patients treated either with ADT monotherapy or with ADT+docetaxel”. Reasons for grouping relied in the superiority of both ADT+ARPI and ADT+ARPI+Docetaxel compared to ADT+Docetaxel or ADT monotherapy,. The aim was to explore if AVPC-alt status could effectively identify which patients benefit more from “intensification strategies”.

The results are presented as hazard ratios (HR) with 95% confidence intervals (CI95%). All statistical analyses were carried out using R statistical software version 4.4.1 and Jamovi statistical software version 2.5.6.

Method S9 - Exploratory analysis and sensitivity analysis:

Exploratory analysis were performed:

- To test AVPC-TSG status impact in de novo and metachronous mHSPC separately.
- MV cox analysis were performed to assess AVPC-TSG status together with all the clinical and demographical variables which included: presence of liver metastasis, presence of lung metastasis, oligometastatic disease, age at mHSPC diagnosis.
- To test the impact of first line ARPI across the patients subgroup as defined by the integrations of AVPC-TSG status with clinical variables (CHAARTED volume criteria, type of disease presentation, ISUP Grade).

Sensitivity analysis:

We conducted sensitivity analysis by creating a multivariate Cox regression model for PFS and OS with AVPC-TSG status variable and each of the individual clinical variables studied.

Results S1 – Modified ESMO-GROW flowchart for real-world evidence studies in oncology

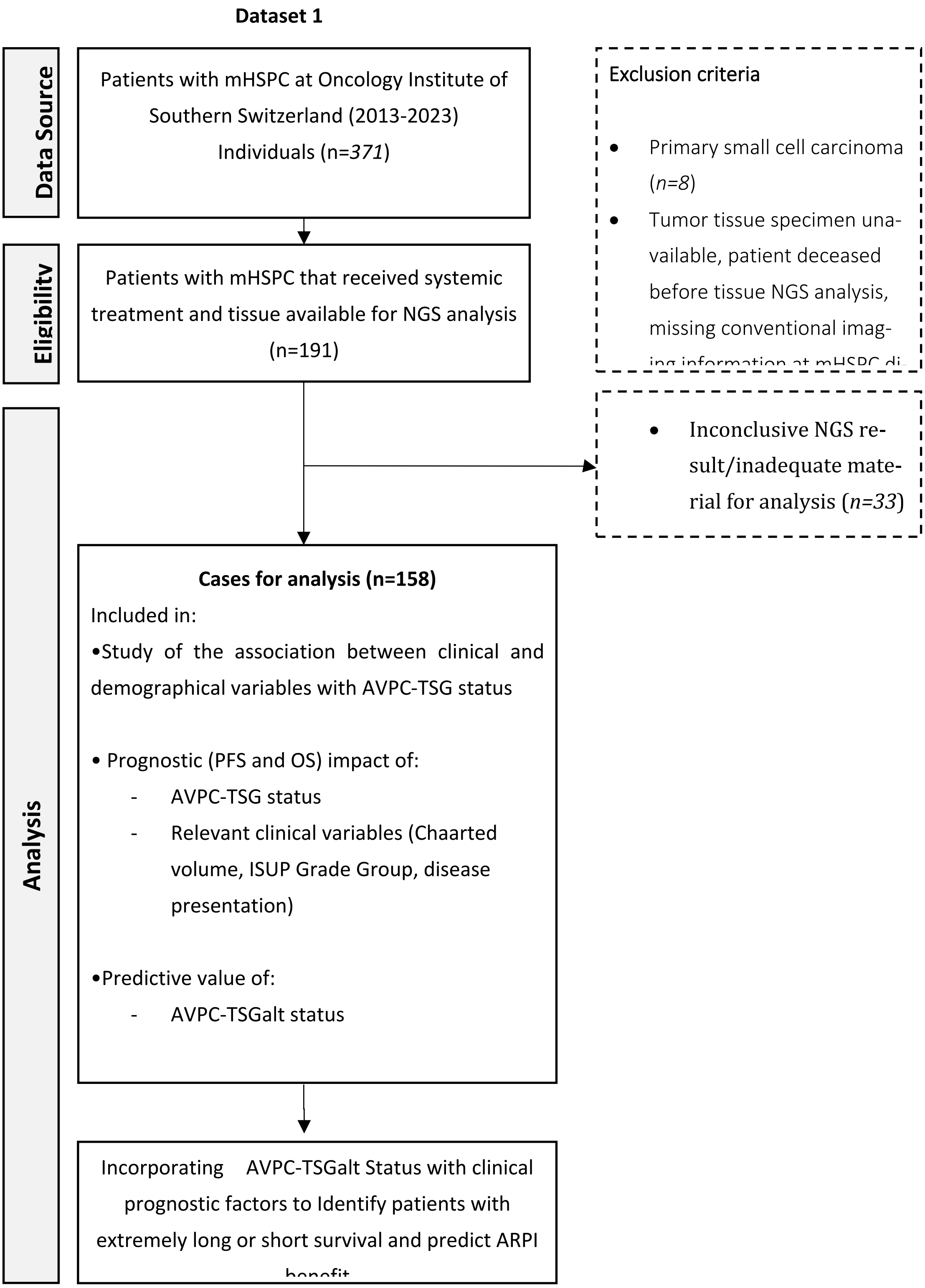

Abbreviations: mHSPC = metastatic hormone-sensitive prostate cancer; TSG= tumor suppressor genes; TSGalt=tumor suppressor genes alterations; AVPC-TSG=aggressive variant prostate cancer associated TSG (TP53, RB1, PTEN); PC= prostate cancer; NGS= Next Generation Sequencing; ISUP= International Society of Urological Pathology.

Table S1 - Site of Analysed Tissue Samples.

Site	***N (%)*** ***Total Number =158***
Radical prostatectomies, prostate biopsies, or transurethral resection of the prostate specimens	*117 (74%)*
lymph nodes	*12 (7.6%)*
Liver	*10 (6.4%)*
Bone	*9 (5.6%)*
Other (lung + soft tissue + peritoneum)	*10 (6.4%)*

Table S2. Type and Frequency of detected gene alterations in the overall population.

Type of Gene Alteration	*Frequency (N=158)*
PTEN/PI3K/AKT pathway alteration	*20 (12.7%)*
Frameshift	*5*
Nonsense	*5*
Missense	*6*
Indel	*2*
Copy Number Gain	*2*
TP53 Alteration	*47 (29.7%)*
Frameshift	*8*
Missense	*27*
Nonsense	*7*
Indel	*2*
Splice site	*3*
RB1 Alteration	*3 (1,9%)*
Frameshift	*1*
Indel	*1*
Nonsense	*1*

Table S3. – Univariate and multivariate Cox proportional hazard models for PFS including site of metastasis and oligometastatic disease status.

Prognostic variable	Levels	Univariate analysis	Multivariate analysis
**Total N. 158**		**HR (95% CI), P-value**	**HR (95% CI), P-value**
**Chaarted Volume**	High Volume	-	-
	Low Volume	0,57 (0.37–0.88) p=0.012**	0,87 (0.53-1.42) p=0.6
**De Novo / Metacronous**	De novo	-	-
	Metacronous	0,89 (0.58–1.36) p= 0.576	
**Liver metastasis**	0	-	-
	1	4,32 (1.98–9.45) p<0.001**	4,28 (1.89-9.68) p<0.001**
**Lung Metastasis**	0	-	-
	1	2,14 (1.20–3.80), p=0.01**	1,79 (0.96-3.35) p=0.068
**Oligometastatic**	0	-	-
	1	0,51 (0.29–0.92) p=0.025*	0,5 (0.27-0.95) p=0.034**
**AVPC-TSG status**	AVPC-TSGalt	-	-
	AVPC-TSGwt	0,57 (0.38–0.87) p=0.01**	0,55 (0.35-0.86) p=0.008**
**ISUP Grade**	<5	-	-
	5	1,22 (0.43–3.44) p=0.703	
**Age at mHSPC**	<75	-	-
	>=75	0,87(0.57–1.32) p=0.506	
**Test for interaction**			
**Chaarted Volume * AVPC-TSG status**	**Df, Chi-square, P-value** 1, 0.013, 0.9713		
**Liver Metastasis * AVPC-TSG status**	**Df, Chi-square, P-value** 1, 0.0492, 0.8244		
**Oligometastatic * AVPC-TSG status**	**Df, Chi-square, P-value** 1, 0.0244, 0.8758		

Abbreviations: PFS= progression free survival; CI = confidence interval; HR = hazard ratio; ISUP=International Society of Urological Pathology; AVPC-TSG= Aggressive variant prostate cancer associated tumor suppressor genes; mHSPC= Metastatic hormone sensitive prostate cancer.

Prognostic variables were included in the multivariable model if P-value ≤0.10

MV model in overall population included: CHAARTED volume, Liver and Lung metastasis, Oligometastatic, TSG status.

Prognostic variables included in the multivariable model were retained statistically significant if P-value ≤0.05 (**).

Table S4. - Univariate and multivariate Cox proportional hazard models for OS including site of metastasis and oligometastatic disease status.

Prognostic variable	Levels	Univariate analysis	Multivariate analysis
**Total N. 158**		**HR (95% CI), P-value**	**HR (95% CI), P-value**
**Chaarted Volume**	High Volume	-	-
	Low Volume	0,37 (0.19–0.72) p=0,003**	1.01 (0.45 -2.26) p>0.9
**Disease Presentation**	De novo	-	-
	Metacronous	0,40 (0.21–0.77) p=0,004**	0.43(0.20-0.94)p=0.035**
**Liver metastasis**	0	-	-
	1	3,33 (1.18–9.40) p=0,023**	4.36 (1.32- 14.3) p=0.015**
**Lung Metastasis**	0	-	-
	1	2,14 (1.02–4.47) p=0,044**	1.67(0.75-3.74)p=0.2
**Oligometastatic**	0	-	-
	1	0,23 (0.07–0.76) p=0,016**	0.27(0.08- 0.94)p=0.040**
**AVPC-TSG status**	AVPC-TSGalt	-	-
	AVPC-TSGwt	0,47 (0.26–0.87) p=0,017**	0.43(0.22- 0.85)p=0.015**
**ISUP Grade**	<5	-	-
	5	1,76 (0.95–3.25) p=0,072*	2.08 (1.09-3.96) p=0.027**
**Age at Mhspc**	<75	-	-
	>=75	0,96 (0.52–1.78) p=0,9	
**Test for interaction**			
**Chaarted Volume * AVPC-TSG status**		**Df, Chi-square, P-value**<br>1, 0.4419, 0.5062	
**Liver Metastasis * AVPC-TSG status**		**Df, Chi-square, P-value**<br>1, 0.688, 0.4069	
**Oligometastatic * AVPC-TSG status**		**Df, Chi-square, P-value**<br>1, 0.003, 0.956	
**Disease Presentation * AVPC-TSG status**		**Df, Chi-square, P-value**<br>1, 0.241, 0.6235	
**ISUP Grade * AVPC-TSG status**		**Df, Chi-square, P-value**<br>1, 4e-04, 0.985	

Abbreviations: OS= overall survival (from treatment start to death); CI = confidence interval; HR = hazard ratio; ISUP=International Society of Urological Pathology; AVPC-TSG= Aggressive variant prostate cancer associated tumor suppressor genes; mHSPC= Metastatic hormone sensitive prostate cancer.
Prognostic variables were included in the multivariable model if P-value ≤0.10*
MV model in overall population included: CHAARTED volume, Liver and Lung metastasis, Oligometastatic, TSG status, disease presentation, ISUP Grade.
Prognostic variables included in the multivariable model were retained statistically significant if P-value ≤0.05 (**).

Table S5. Sensitivity Analysis for PFS: Univariate and multivariate Cox proportional hazard models for PFS.

Prognostic variable	Levels	Univariate analysis	Multivariate analysis
**Total N. 158**		**HR (95% CI), P-value**	**HR (95% CI), P-value**
**MV with CHAARTED volume criteria**			
AVPC-TSG status	AVPC-TSGalt	-	-
	AVPC-TSGwt	0,57 (0.38–0.87) p=0.01**	0.54 (0.35-0.83, p=0.005)**
Chaarted Volume	High Volume	-	-
	Low Volume	0,57 (0.37–0.88) p=0.012**	0.58 (0.37-0.90, p=0.014)**
**MV with liver metastasis**			
AVPC-TSG status	AVPC-TSGalt	-	-
	AVPC-TSGwt	0,57 (0.38–0.87) p=0.01**	0.54 (0.35-0.83, p=0.005)**
Liver metastasis	0	-	-
	1	4,32 (1.98–9.45) p<0.001**	4.93 (2.24-10.87, p<0.001)**
**MV with Lung Metastasis**			
AVPC-TSG status	AVPC-TSGalt	-	-
	AVPC-TSGwt	0,57 (0.38–0.87) p=0.01**	0.63 (0.41-0.98, p=0.043)**
Lung Metastasis	0	-	-
	1	2,14 (1.20–3.80), p=0.01**	1.77 (0.97-3.24, p=0.063)
**MV with Oligometastatic**			
AVPC-TSG status	AVPC-TSGalt	-	-
	AVPC-TSGwt	0,57 (0.38–0.87) p=0.01**	0.53 (0.34-0.81, p=0.003)**
Oligometastatic	0	-	-
	1	0,51 (0.29–0.92) p=0.025**	0.46 (0.26-0.83, p=0.010)**

Abbreviations: PFS= progression free survival; CI = confidence interval; HR = hazard ratio; ISUP=International Society of Urological Pathology; AVPC-TSG= Aggressive variant prostate cancer associated tumor suppressor genes; mHSPC= Metastatic hormone sensitive prostate cancer.

Prognostic variables were included in the multivariable model if P-value ≤0.10*

MV model were created to assess TSG status impact with each clinical prognostic variable: CHAARTED volume, Liver and Lung metastasis, Oligometastatic.

Prognostic variables included in the multivariable model were retained statistically significant if P-value ≤0.05 (**).

Table S6. - Sensitivity Analysis for OS: Univariate and multivariate Cox proportional hazard models for OS.

Prognostic variable	Levels	Univariate analysis	Multivariate analysis
**Total N. 158**		**HR (95% CI), P-value**	**HR (95% CI), P-value**
**MV with CHAARTED Volume Criteria**			
AVPC-TSG status	AVPC-TSGalt	-	-
	AVPC-TSGwt	0,47 (0.26–0.87) p=0,017**	0.53 (0.29-0.99), p=0.045**
Chaarted Volume	High Volume	-	-
	Low Volume	0,37 (0.19–0.72) p=0,003**	0.41 (0.21-0.79), p=0.008**
**MV with Disease Presentation**			
AVPC-TSG status	AVPC-TSGalt	-	-
	AVPC-TSGwt	0,47 (0.26–0.87) p=0,017**	0.53 (0.29-0.99), p=0.045**
Disease Presentation	De novo	-	-
	Metacronous	0,40 (0.21–0.77) p=0,004**	0.40 (0.21-0.79), p=0.009**
**MV with Liver Metastasis**			
AVPC-TSG status	AVPC-TSGalt	-	-
	AVPC-TSGwt	0,47 (0.26–0.87) p=0,017**	0.41(0.21- 0.77)p=0.006*
Liver metastasis	0	-	-
	1	3,33 (1.18–9.40) p=0,023**	4.80 (1.62- 14.22) p=0.005**
**MV with Lung Metastasis**			
AVPC-TSG status	AVPC-TSGalt	-	-
	AVPC-TSGwt	0,47 (0.26–0.87) p=0,017**	0.53(0.28- 1.03)p=0.063*
Lung Metastasis	0	-	-
	1	2,14 (1.02–4.47) p=0,044**	1.57(0.71-3.49)p=0.269
**MV with Oligometastatic**			
AVPC-TSG status	AVPC-TSGalt	-	-
	AVPC-TSGwt	0,47 (0.26–0.87) p=0,017**	0.45(0.24- 0.83)p=0.011*
Oligometastatic	0	-	-
	1	0,23 (0.07–0.76) p=0,016**	0.22(0.07- 0.72)p=0.013**
**MV with ISUP Grade**			
AVPC-TSG status	AVPC-TSGalt	-	-
	AVPC-TSGwt	0,47 (0.26–0.87) p=0,017**	0.41(0.22- 0.76)p=0.005*
ISUP Grade	<5	-	-
	5	1,76 (0.95–3.25) p=0,072*	2.03 (1.08-3.83) p=0.028*

Abbreviations: rPFS= radiological progression free survival; CI = confidence interval; HR = hazard ratio; ISUP=International Society of Urological Pathology;
Prognostic variables were included in the multivariable model if P-value ≤0.10*
MV model were created to assess TSG status impact with each clinical prognostic variable: CHAARTED volume, Liver and Lung metastasis, Oligometastatic, TSG status, disease presentation, ISUP Grade..
Prognostic variables included in the multivariable model were retained statistically significant if P-value ≤0.05 (**).

Table S7. – Exploratory Analysis: Univariate and multivariate Cox proportional hazard models for PFS and OS based on Disease Presentation.

Prognostic variable	Levels	Univariate analysis
**Total N. 100 De Novo mHSPC**		**HR (95% CI), P-value**
PFS- AVPC-TSG status	AVPC-TSGalt	-
	AVPC-TSGwt	0.53 (0.31-0.91), p=0.020**
OS- AVPC-TSG status	AVPC-TSGalt	-
	AVPC-TSGwt	0.53 (0.25-1.12, p=0.096*
**Total N. 58 Relapsed mHSPC**		**HR (95% CI), P-value**
PFS- AVPC-TSG status	AVPC-TSGalt	-
	AVPC-TSGwt	0.61 (0.30-1.24), p=0.169
OS- AVPC-TSG status	AVPC-TSGalt	-
	AVPC-TSGwt	0.47 (0.15-1.44), p=0.185

Abbreviations: PFS= progression free survival; CI = confidence interval; HR = hazard ratio; ISUP=International Society of Urological Pathology; mHSPC= metastatic hormone sensitive prostate cancer; AVPC-TSG=Aggressive variant prostate cancer associated tumor suppressor genes
Prognostic variables were considered statistically significant in if P-value ≤0.05**

Table S8. Exploratory Analysis: Univariate Cox proportional hazard models for PFS and OS based on AVPC-TSGalt number.

Prognostic variable	Levels	Univariate analysis
**Total N. 158**		**HR (95% CI), P-value**
PFS- AVPC-TSG status	AVPC-TSGwt	-
	AVPC-TSG 1 alteration	1.71 (1.11-2.64), p=0.015**
	AVPC-TSG 2 or 3 alterations	2.10 (0.75-5.85), p=0.158
OS- AVPC-TSG status	AVPC-TSGwt	-
	AVPC-TSG 1 alteration	2.01 (1.07-3.79), p=0.031**
	AVPC-TSG 2 or 3 alterations	3.37 (0.98-11.58), p=0.053

Abbreviations: PFS= progression free survival; CI = confidence interval; HR = hazard ratio; mHSPC= metastatic hormone sensitive prostate cancer; AVPC-TSG=Aggressive variant prostate cancer associated tumor suppressor genes, AVPC-TSG 1 alteration= presence of 1 single alteration in TP53 gene, RB1 gene, PTEN/PI3K/AKT genes; AVPC-TSG 2-3 alterations= presence of at least 2 alterations in TP53 gene, RB1 gene, PTEN/PI3K/AKT genes.
Prognostic variables were considered statistically significant in if P-value ≤0.05**

Table S9 – Integrating AVPC-TSG Status with Chaarted volume criteria: Univariate Cox Analysis for PFS and OS and Pairwise Comparison

Progression Free Survival

Cox Table: Chaarted volume criteria * AVPC-TSG status

Explanatory	Levels	All	HR (Univariable)
Chaarted volume criteria * AVPC-TSG status	TSGwt + Low Volume	43 (27.2)	-
	TSGalt + High Volume	36 (22.8)	2.98 (1.63-5.46, p<0.001)
	TSGalt or High Volume	79 (50.0)	1.74 (1.00-3.03, p=0.050)

Pairwise Comparisons: Chaarted volume criteria * AVPC-TSG status

Levels	Levels	p-value
TSGalt + High Volume	TSGwt + Low Volume	0.001
TSGalt or High Volume	TSGwt + Low Volume	0.049
TSGalt or High Volume	TSGmut + High Volume	0.049

Note. p-value adjustement method: holm

Overall Survival

Cox Table: Chaarted volume criteria * AVPC-TSG status

Explanatory	Levels	All	HR (Univariable)
Chaarted volume criteria * AVPC-TSG status	TSGwt + Low Volume	43 (27.2)	-
	TSGalt + High Volume	36 (22.8)	5.42 (2.07-14.20, p=0.001)
	TSGalt or High Volume	79 (50.0)	3.50 (1.37-8.98, p=0.009)

Pairwise Comparisons: Chaarted volume criteria * AVPC-TSG status

Levels	Levels	p-value
TSGalt + High Volume	TSGwt + Low Volume	< .001
TSGalt or High Volume	TSGwt + Low Volume	0.022
TSGalt or High Volume	TSGmut + High Volume	0.253

Note. p-value adjustement method: holm

Abbreviations: PFS= progression free survival; OS=Overall survival; CI = confidence interval; HR = hazard ratio; ISUP=International Society of Urological Pathology; mHSPC= metastatic hormone sensitive prostate cancer; AVPC-

TSG=Aggressive variant prostate cancer associated tumor suppressor genes. Prognostic variables were considered statistically significant in if P-value ≤0.05**

Table S10. Type of I Line Treatment Based on AVPC-TSG Status and Chaarted Disease Volume Status.

Variable	N	AVPC-TSGwt+LV (N=43)	AVPC-TSGalt or HV (N=79)	AVPC-TSGalt+HV (N=36)	Test Statistic Pearson Test
**I line mHSPC treatment**	**158**				*P=0.08*[1]
ADT		19/43 (44.2%)	29/79 (36.8%)	7/36 (19.4%)	
ADT+ARPI		22/43 (51.1%)	36/79 (45.6%)	19/36 (52,8%)	
ADT+Docetaxel		2/43 (4.7%)	7/79 (8.9%)	6/36 (16.7%)	
ADT+ARPI+Docetaxel		0/43 (0%)	7/79 (8.9%)	4/36 (11.1%)	
**Received ARPI in I line mHSPC**	**158**	22/43 (51.1%)	43/79 (54.4%)	23/36 (63.9%)	*P=0.50*[1]

Abbreviations: mHSPC= metastatic hormone sensitive prostate cancer; AVPC-TSG=Aggressive variant prostate cancer associated tumor suppressor genes; HV= high volume; LV=Low volume; ARPI= Androgen receptor pathway inhibitors; ADT=Androgen deprivation therapy N is the number of non-missing value. [1]Pearson test.

Table S11. Univariate Cox proportional hazard models for PFS and OS comparing "Intermediate Risk Subgroup" components (AVPC-TSGalt/LV VS AVPC-TSGwt/HV).

**Prognostic variable**	**Levels**	**Pts N (%)**	***Univariate Analysis***
**Total pts N. 79**<br>**PFS Analysis**			**HR (95% CI), P-value**
Intermediate Risk Subgroub	AVPC-TSGwt/HV	52 (65.8)	-
	AVPC-TSGalt/LV	27 (34.2)	0.98 (0.52-1.87), p=0.954
**Total pts N. 79**<br>**OS Analysis**			**HR (95% CI), P-value**
Intermediate Risk Subgroub	AVPC-TSGwt/HV	52 (65.8)	-
	AVPC-TSGalt/LV	27 (34.2)	0.84 (0.34-2.08), p=0.706
Abbreviations: PFS= progression free survival; CI = confidence interval; HR = hazard ratio; Pts N= total number of patients included in the analysis; AVPC=Aggressive variant prostate cancer; TSG= Tumor suppressor genes (TP53, RB1 or PTEN); mHSPC= metastatic hormone sensitive prostate cancer; ARPI= Androgen receptor pathway inhibitors; ADT=Androgen deprivation therapy; UV=Univariate; AVPC=Aggressive-Variant Prostate Cancer<br>Variables were retained statistically significant if P-value ≤0.05 (**).			

Table S12. – Integrating AVPC-TSG Status with ISUP Grade: Univariate Cox Analysis for PFS and OS and Pairwise Comparison.

Progression Free Survival

Cox Table: ISUP Grade * AVPC-TSG Status

Explanatory	Levels	All	HR (Univariable)
ISUP Grade * AVPC-TSG Status	TSGalt+ISUP5	31 (19.6)	-
	TSGwt+ISUP<5	46 (29.1)	0.41 (0.22-0.79, p=0.008)
	TSGalt or ISUP5	81 (51.3)	0.73 (0.43-1.25, p=0.257)

Pairwise Comparisons: ISUP Grade * AVPC-TSG Status

Levels	Levels	p-value
TSGwt+ISUP<5	TSGalt+ISUP5	0.034
TSGalt or ISUP5	TSGalt+ISUP5	0.257
TSGalt or ISUP5	TSGwt+ISUP<5	0.056

Note. p-value adjustement method: holm

Overall Survival

Cox Table: ISUP Grade * AVPC-TSG Status

Explanatory	Levels	All	HR (Univariable)
ISUP Grade * AVPC-TSG Status	TSGalt+ISUP5	31 (19.6)	-
	TSGwt+ISUP<5	46 (29.1)	0.20 (0.07-0.53, p=0.001)
	TSGalt or ISUP5	81 (51.3)	0.43 (0.21-0.86, p=0.017)

Pairwise Comparisons: ISUP Grade * AVPC-TSG Status

Levels	Levels	p-value
TSGwt+ISUP<5	TSGalt+ISUP5	0.006
TSGalt or ISUP5	TSGalt+ISUP5	0.023
TSGalt or ISUP5	TSGwt+ISUP<5	0.087

Cox Table: ISUP Grade * AVPC-TSG Status

Explanatory	Levels	All	HR (Univariable)

Note. p-value adjustement method: holm

Abbreviations: PFS= progression free survival; OS=Overall survival; CI = confidence interval; HR = hazard ratio; ISUP=International Society of Urological Pathology; mHSPC= metastatic hormone sensitive prostate cancer; AVPC-TSG=Aggressive variant prostate cancer associated tumor suppressor genes. Prognostic variables were considered statistically significant in if P-value ≤0.05**

Table S13. – Integrating AVPC-TSG Status with Disease Presentation: Univariate Cox Analysis for PFS and OS and Pairwise Comparison.

Progression Free Survival:

Cox Table: Disease Presentation * AVPC-TSG Status

Explanatory	Levels	All	HR (Univariable)
Disease Presentation * AVPC-TSG Status	TSGalt+De Novo	43 (27.2)	-
	TSGalt+ Relapsed	38 (24.1)	0.58 (0.34-1.01, p=0.054)
	TSGalt or De Novo	77 (48.7)	0.59 (0.36-0.96, p=0.035)

Pairwise Comparisons: Disease Presentation * AVPC-TSG Status

Levels	Levels	p-value
TSGwt+Relapsed	TSGalt+DeNovo	0.100
TSGalt or De Novo	TSGalt+DeNovo	0.098
TSGalt or De Novo	TSGwt+Relapsed	0.930

Note. p-value adjustement method: holm

Overall Survival:

Cox Table: Disease Presentation * AVPC-TSG Status

Explanatory	Levels	All	HR (Univariable)
Disease Presentation * AVPC-TSG Status	TSGalt+De Novo	43 (27.2)	-
	TSGalt+ Relapsed	38 (24.1)	0.23 (0.10-0.55, p=0.001)
	TSGalt or De Novo	77 (48.7)	0.47 (0.24-0.93, p=0.030)

Pairwise Comparisons: Disease Presentation * AVPC-TSG Status

Levels	Levels	p-value
TSGwt+Relapsed	TSGalt+DeNovo	< .001
TSGalt or De Novo	TSGalt+DeNovo	0.069
TSGalt or De Novo	TSGwt+Relapsed	0.113

Cox Table: Disease Presentation * AVPC-TSG Status

Explanatory	Levels	All	HR (Univariable)

Note. p-value adjustement method: holm

Abbreviations: PFS= progression free survival; OS=Overall survival; CI = confidence interval; HR = hazard ratio; ISUP=International Society of Urological Pathology; mHSPC= metastatic hormone sensitive prostate cancer; AVPC-TSG=Aggressive variant prostate cancer associated tumor suppressor genes. Prognostic variables were considered statistically significant in if P-value ≤0.05**

Table S14. – Integrating disease presentation and ISUP Grade Group with Chaarted volume criteria: Univariate Cox Analysis for PFS and Pairwise Comparison.

Prognostic variable	Levels	Pts N (%)	Univariate analysis
**Total pts N. 158<br>PFS by Disease Volume and Presentation**			**HR (95% CI), P-value**
	High Volume AND De Novo	71 (44.9)	-
	Low Volume AND Metachronous	41 (25.9)	0.67 (0.40-1.10), p=0.115
	High Volume OR De Novo	46 (29.1)	0.58 (0.35-0.98), p=0.041*
**Subgroup Comparison (Holm)**			
**Levels compared**			**p-value**
High Volume OR De Novo **VS** Low Volume AND Metachronous			0.620
High Volume OR De Novo **VS** High Volume AND De Novo			0.208
High Volume AND De Novo **VS** Low Volume AND Metachronous			0.208
**Total pts N. 63<br>PFS by Disease Volume and ISUP Grade Group**			**HR (95% CI), P-value**
	High Volume AND ISUP Grade Group 5	44 (27.8)	-
	Low Volume AND ISUP Grade Group < 5	34 (21.5)	0.44 (0.24-0.81), p=0.009**
	High Volume OR ISUP Grade Group 5	80 (50.6)	0.71 (0.44-1.14), p=0.155
**Subgroup Comparison (Holm)**			

**Levels compared**	**p-value**
High Volume OR ISUP Grade Group 5 **VS** Low Volume AND ISUP Grade Group <5	0.189
High Volume OR ISUP Grade Group 5 **VS** High Volume AND ISUP Grade Group 5	0.189
High Volume AND ISUP Grade Group 5 **VS** Low Volume AND ISUP Grade Group <5	0.032**
Abbreviations: PFS= progression free survival; CI = confidence interval; HR = hazard ratio; Pts N= total number of patients included in the analysis; AVPC=Aggressive variant prostate cancer; TSG= Tumor suppressor genes (TP53, RB1 or PTEN); mHSPC= metastatic hormone sensitive prostate cancer; ARPI= Androgen receptor pathway inhibitors; ADT=Androgen deprivation therapy; MV=Multivariate<br>Variables were retained statistically significant if P-value ≤0.05 (**).	

Table S15. Univariate and Multivariate Cox proportional hazard models for PFS based on first line treatment in the overall population and in AVPC-TSGalt and AVPC-TSGwt patients.

**Prognostic variable**	**Levels**	**Pts N (%)**	**Univariate analysis**
**Total pts N. 158**<br>**PFS in the Overall Population**			**HR (95% CI), P-value**
First line treatment	ADT or ADT+Docetaxel	70 (44.3)	-
	ADT+ARPI or ADT+ARPI+Docetaxel	88 (55.7)	0.64 (0.41-0.99), p=0.044**
**Total pts N. 63**<br>**PFS in AVPC-TSGalt patients**			**HR (95% CI), P-value**
First line treatment	ADT or ADT+Docetaxel	31 (49.2)	-
	ADT+ARPI or ADT+ARPI+Docetaxel	32 (50.8)	1.13 (0.58-2.19), p=0.721
**Total pts N. 95**<br>**PFS in AVPC-TSGwt patients**			**HR (95% CI), P-value**
First line treatment	ADT or ADT+Docetaxel	39 (41.1)	-
	ADT+ARPI or ADT+ARPI+Docetaxel	56 (58.9)	0.51 (0.28-0.93), p=0.029**
**Multivariate cox analysis for PFS between "First line treatment" and "AVPC-TSG status"**			
**First line treatment**			**MV HR (95% CI), P-value**
	ADT or ADT+Docetaxel		-
	ADT+ARPI or ADT+ARPI+Docetaxel		0.70 (0.45-1.10), p=0.127
**AVPC-TSG Status**	AVPC-TSGalt		-
	AVPC-TSGwt		0.62 (0.40-0.95), p=0.028**
**Test for interaction**			
**First line treatment * AVPC-TSG status**	**Df, Chi-square, P-value**<br>1, 2.6468, 0.1038		

Abbreviations: PFS= progression free survival; CI = confidence interval; HR = hazard ratio; Pts N= total number of patients included in the analysis; AVPC=Aggressive variant prostate cancer; TSG= Tumor suppressor genes (TP53, RB1 or PTEN); mHSPC= metastatic hormone sensitive prostate cancer; ARPI= Androgen receptor pathway inhibitors; ADT=Androgen deprivation therapy; MV=Multivariate
Variables were retained statistically significant if P-value ≤0.05 (**).

Table S16. Exploratory Analysis: Univariate and Multivariate Cox proportional hazard models for PFS based on first line treatment in subgroups defined by AVPC-TSG status and CHAARTED volume criteria.

Prognostic variable	Levels	Pts N (%)	Univariate analysis
**Total pts N. 43 PFS in "AVPC-TSGwt+LV" patients**			**HR (95% CI), P-value**
First line treatment	ADT or ADT+Docetaxel	21 (48.8)	-
	ADT+ARPI or ADT+ARPI+Docetaxel	22 (51.2)	0.98 (0.37-2.57), p=0.959
**Total pts N. 63 PFS in "AVPC-TSGalt+HV" patients**			**HR (95% CI), P-value**
First line treatment	ADT or ADT+Docetaxel	13 (36.1)	-
	ADT+ARPI or ADT+ARPI+Docetaxel	23 (63.9)	1.07 (0.47-2.40), p=0.873
**Total pts N. 95 PFS in "AVPC-TSGalt or HV" patients**			**HR (95% CI), P-value**
First line treatment	ADT or ADT+Docetaxel	36 (45.6)	-
	ADT+ARPI or ADT+ARPI+Docetaxel	43 (54.4)	0.36 (0.19-0.70), p=0.002**
**Multivariate cox analysis for PFS between "First line treatment" and "AVPC-TSG status/Chaarted Volume Criteria"**			
			**MV HR (95% CI), P-value**
**First line treatment**	*ADT or ADT+Docetaxel*		-
	ADT+ARPI or ADT+ARPI+Docetaxel		0.61 (0.39-0.95), p=0.028**
**AVPC-TSG status/Chaarted Volume Criteria**	AVPC-TSGwt+LV		-
	AVPC-TSGalt or HV		1.84 (1.06-3.20), p=0.031**
	AVPC-TSGalt+HV		3.07 (1.67-5.61), p<0.001**
**Test for interaction**			
**First line treatment * AVPC-TSG status/Chaarted Volume Criteria**			**Df, Chi-square, P-value** 2, 5.1566 , 0.0759

Abbreviations: PFS= progression free survival; CI = confidence interval; HR = hazard ratio; Pts N= total number of patients included in the analysis; AVPC=Aggressive variant prostate cancer; TSG= Tumor suppressor genes (TP53, RB1 or PTEN); mHSPC= metastatic hormone sensitive prostate cancer; ARPI= Androgen receptor pathway inhibitors; ADT=Androgen deprivation therapy; AVPC-TSG status/Chaarted Volume Criteria= AVPC-TSG Status integrated with Chaarted volume criteria.
Variables were retained statistically significant if P-value ≤0.05 (**).

Table S17. Exploratory Analysis: Univariate Cox proportional hazard models for PFS based on first line treatment in the "intermediate risk subgroup" components.

Prognostic variable	Levels	Pts N (%)	*Univariate Analysis*
	**Total pts N. 52 PFS Analysis**		**HR (95% CI), P-value**
AVPC-TSGwt/HV	ADT or ADT+Docetaxel	18 (34.6)	-
	ADT+ARPI or ADT+ARPI+Docetaxel	34 (65.4)	0.25 (0.12-0.54), p<0.001**
	**Total pts N. 27 PFS Analysis**		**HR (95% CI), P-value**
AVPC-TSGalt/LV	ADT or ADT+Docetaxel	18 (66.7)	-
	ADT+ARPI or ADT+ARPI+Docetaxel	9 (33.3)	0.59 (0.13-2.71), p=0.500

Abbreviations: PFS= progression free survival; CI = confidence interval; HR = hazard ratio; Pts N= total number of patients included in the analysis; AVPC=Aggressive variant prostate cancer; TSG= Tumor suppressor genes (TP53, RB1 or PTEN); mHSPC= metastatic hormone sensitive prostate cancer; ARPI= Androgen receptor pathway inhibitors; ADT=Androgen deprivation therapy; UV=Univariate; AVPC=Aggressive-Variant Prostate Cancer
Variables were retained statistically significant if P-value ≤0.05 (**).

Table S18. Self-reported ESMO-GROW informative Score

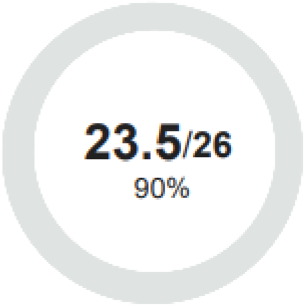

23/35 Yes, fully reported

1/35 Yes, partially reported

2/35 Not reported

9/35 Not applicable

**ESMO-GROW Checklist for the study entitled: Prognostic Implications of Early Treatment-Emergent Hypertension in de Novo Metastatic Hormone-Sensitive Prostate Cancer: A Retrospective Real-World Analysis**	Yes, fully reported	Yes, partially reported	Not reported	Not applicable
**1: Title**				
1.1: Concisely include relevant key terms referring to the study type, study population, objectives, data sources and outcomes, depending on the study. Consider including the terms 'real-world' or 'observational'	●			
**2: Introduction**				
2.1: Explain the scientific rationale for the research question(s), providing concise background information on previous core evidence from systematic reviews, meta-analyses, clinical trials and/or real-world evidence studies	●			
2.2: Identify the gaps in evidence and explain why and how they can be suitably addressed by real-world evidence research. Specify the new evidence that is expected from the current study	●			
2.3: Briefly introduce the aim(s) of the study	●			
**3: Methods**				
**Study objective(s), design, data sources and variables**				
3.1: Provide the study research question(s) including a description of the patients or the object under study and the target outcome(s)	●			
3.2: Provide the study objective(s) and consider classifying the type of research as descriptive and/or analytical (explanatory or predictive)	●			
3.3: Provide relevant information to describe and classify the study design used to address the research question	●			
3.4: Give a clear definition of the eligibility criteria used to select the patients or objects under study, particularly regarding cancer-related aspects	●			
3.5: Report the specific type and purpose of real-world data source(s) used, providing a detailed description and the reason(s) why the source was considered appropriate for the study objectives	●			
3.6: When multiple real-world data sources are used, provide details on interoperability, including identification of duplicated cases or data linkage from separate databases				●
3.7: Provide details and timings of source and study data management. Consider specifying methods of raw data collection, updates and completeness, data extraction, cleaning and/or quality controls and validation		●		
3.8: Provide core details on database and/or study registration, governance, ownership, metadata and full data accessibility in the main text or supplementary material			●	
3.9: Identify the data source of each core variable, its definition, if the variable was derived or coded, and describe how the derivation or coding was conducted and validated	●			
3.10: Specify the time points of core variables in relation to the cancer disease trajectory	●			
3.11: Provide a complete list of core variables included in the study. Variables can be grouped as baseline characteristics, exposure, and outcomes or endpoints	●			
3.12: For biomarker-related studies, provide details on biomarker description, timing, and methods of assessment and analytical validation	●			

**Statistical analysis and artificial intelligence methods**				
3.13: Summarise the main aspects of the statistical analysis	🟢			
3.14: When applicable, provide details on the pre-planned sample size requirements and power of the study				⚫
3.15: Specify the pre-planned strategies to identify and mitigate the main sources of bias	🟢			
3.16: Clearly distinguish prespecified from post hoc analyses, especially for subgroup analyses				⚫
3.17: Provide information on internal and external validity, as well as any sensitivity analyses			🔴	
3.18: For analytical studies, the full version of the statistical analysis plan should be provided in the supplementary material, including a brief explanation of any amendments				⚫
3.19: When applicable, specify which machine learning, deep learning or alternative artificial intelligence method has been used				⚫
3.20: When reporting real-world data analysis with artificial intelligence (e.g. machine learning and deep learning) algorithms, include comprehensive aspects on data pre-processing techniques, feature engineering strategies and model development				⚫
3.21: Address the artificial intelligence model explainability and interpretability, and present the plan for integration into clinical practice, if applicable				⚫
3.22: When applicable, briefly describe the multidisciplinary team required for the study and explain how these needs were met				⚫
**4: Results**				
4.1: Provide the number of cases excluded or nonparticipating and reasons at each stage of sample selection, as well as numbers lost to follow-up. Compare the cases excluded with those included in the analyses. Illustrate this with a flowchart	🟢			
4.2: Describe the baseline characteristics of the cases included (e.g. clinico-demographic and tumour characteristics). The baseline characteristics of different groups under analysis should be compared, if applicable	🟢			
4.3: Report the results of the primary analysis of study outcomes. Briefly describe the results of exploratory analyses if relevant (prespecified and/or post hoc). Provide details of how readers can access the full results	🟢			
**5: Discussion and conclusions**				
**Discussion**				
5.1: Summarise the core results that address the primary research question(s) and objectively discuss the data in relation to the best available evidence on the topic. Avoid a convenient selection of literature to support a point	🟢			
5.2: Discuss the strengths and limitations of the current study, including the main biases, how the strategies applied contributed to bias avoidance or mitigation, and, if applicable, in which direction the authors estimate that residual bias may influence the core results of the study	🟢			
5.3: Discuss the generalisability of the study results and their potential implications for clinical practice, health policies or public health and for the generation of hypotheses for future research	🟢			
**Conclusions**				
5.4: Provide a balanced summary of core results relating to the primary research question and the main implications for clinical practice, health policies and/or public health. Suggest further research considering the remaining unmet needs and limitations from the reported study	🟢			
**6: Final considerations**				
6.1: Specify all relevant study sponsorship(s) as well as direct and/or indirect or in-kind funding				⚫
6.2: Specify all relevant acknowledgements, author disclosures, individual contributions and other final considerations as per journal regulations	🟢			

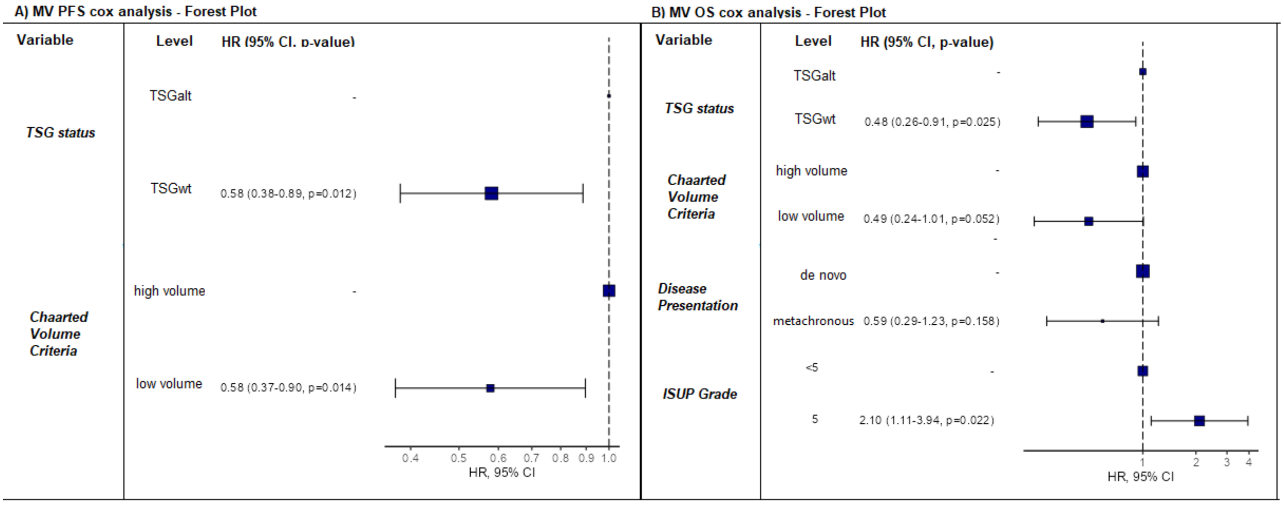

Figure S1. Forest Plot of MV Cox Analysis for PFS and OS with relevant clinical variables.

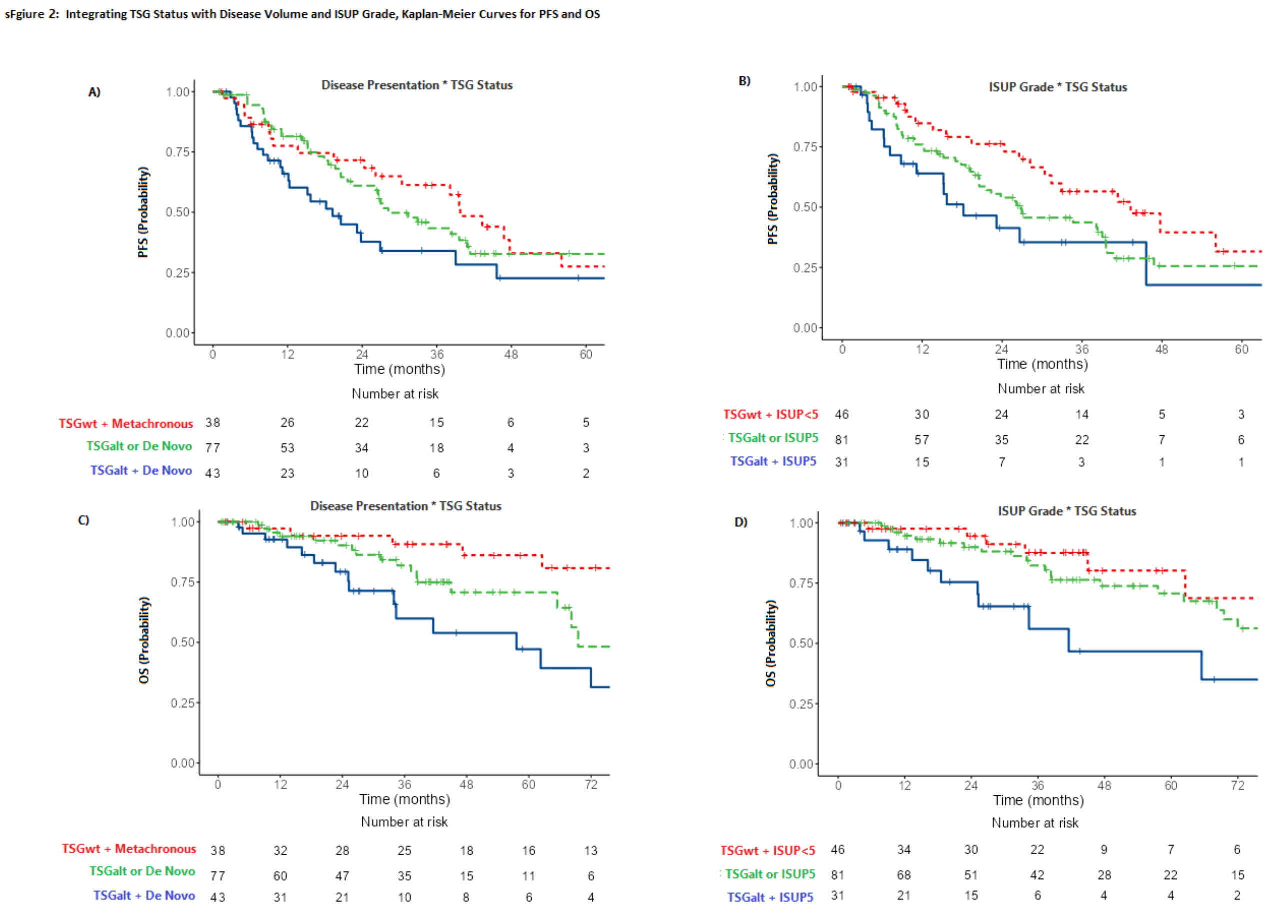

Figure S2. Integrating AVPC-TSG Status with Disease Volume and ISUP Grade, Kaplan-Meier Curves for PFS and OS.
